# Seasonal Effects in a Lake Sediment Archaeal Community of the Brazilian Savanna

**DOI:** 10.1155/2014/957145

**Published:** 2014-07-20

**Authors:** Thiago Rodrigues, Elisa Catão, Mercedes M. C. Bustamante, Betania F. Quirino, Ricardo H. Kruger, Cynthia M. Kyaw

**Affiliations:** ^1^Department of Cell Biology, Biological Sciences Institute, University of Brasília, 70910-900 Brasília, DF, Brazil; ^2^Department of Ecology, Biological Sciences Institute, University of Brasília, 70910-900 Brasília, DF, Brazil; ^3^Department of Genomics Science and Biotechnology, Catholic University of Brasília, 70790-160 Brasília, DF, Brazil; ^4^Embrapa-Agroenergy, 70770-901 Brasília, DF, Brazil

## Abstract

The Cerrado is a biome that corresponds to 24% of Brazil's territory. Only recently microbial communities of this biome have been investigated. Here we describe for the first time the diversity of archaeal communities from freshwater lake sediments of the Cerrado in the dry season and in the transition period between the dry and rainy seasons, when the first rains occur. Gene libraries were constructed, using *Archaea*-specific primers for the 16S rRNA and *amoA* genes. Analysis revealed marked differences between the archaeal communities found in the two seasons. I.1a and I.1c Thaumarchaeota were found in greater numbers in the transition period, while MCG *Archaea* was dominant on the dry season. Methanogens were only found in the dry season. Analysis of 16S rRNA sequences revealed lower diversity on the transition period. We detected archaeal *amoA* sequences in both seasons, but there were more OTUs during the dry season. These sequences were within the same cluster as *Nitrosotalea devanaterra's amoA* gene. The principal coordinate analysis (PCoA) test revealed significant differences between samples from different seasons. These results provide information on archaeal diversity in freshwater lake sediments of the Cerrado and indicates that rain is likely a factor that impacts these communities.

## 1. Introduction

The Brazilian savanna, also known as the Cerrado, corresponds to 24% of the country's territory [1] and is characterized by two defined seasons: the dry season, which occurs from May to September, and the rainy season, which occurs from October to April [2]. Though there are some studies on the diversity of* Archaea* in different Brazilian environments [3–7], little is known about archaeal communities in the Cerrado. Recently, our group described the archaeal richness in Cerrado soils [8], but there are currently no reports on the diversity of* Archaea* in freshwater lake sediments in this biome.

Lake sediments are environments with a high abundance of microorganisms [9], which are subjected to changes in nutrient composition associated with events of resuspension and redeposition of the sediment surface caused by water flow [10]. Rain is one of many different environmental factors that cause these events [11]. Although there are many studies on the diversity of* Archaea* in lake sediments [12–16], there are currently no reports that describe changes in the community caused by the occurrence of rain.

Ammonia oxidation to nitrite has an important role in the global biogeochemical nitrogen cycle [17] and it is known that* Bacteria* and* Archaea* are both capable of ammonia oxidation [18, 19]. Many mesophilic* Archaea* found in soils, water, and freshwater sediments [20], formerly classified as Crenarchaeota, are now considered members of the recently proposed Thaumarchaeota phylum [21], which includes all of the ammonia oxidizing* Archaea* (AOA) known so far [22].

Here we describe for the first time the diversity of archaeal communities from freshwater lake sediments of the Cerrado in the dry season and in the transition period between the dry and rainy seasons, when the first rains occur. Eight gene libraries were obtained and sequenced, four using* Archaea*-specific primers for the 16S rRNA gene and the other four with* amoA* gene primers specific to ammonia-oxidizing* Archaea*. The sequences obtained were analyzed to determine whether there are differences between the communities in lake sediments of different seasons. Our results show two very different community profiles on each season, indicating a possible effect of seasonal change.

## 2. Materials and Methods

### 2.1. Study Site and Sampling

Sediments were obtained from a lake in the “Parque Nacional das Sempre Vivas,” a National Park located in the Serra do Espinhaço, in the state of Minas Gerais, Brazil, within the Cerrado biome. The sediments were sampled in the Baixo Inhancica bay, near the margin and the distance between the sampling spots was 15 m. Samples will be referred here as RIB-A (17°46′16.21078′′S, 43°37′35.5481′′W) and RIB-B (17°46′16.49918′′S, 43°37′35.94635′′W) and were considered replicates. Samples were collected with PVC tubes 10 cm in diameter by introducing the tube into the sediments located near the lake shore. Sediment samples were retrieved from depths between 0 and 10 cm and kept on ice until stored at −20°C.

The dry season samples RIB-A and RIB-B were collected on May 7, 2010, and will therefore be referred to as RIB-A D and RIB-B D. The samples from the transition period between the dry and rainy seasons were RIB-A and RIB-B collected on September 13, 2010, and will be referred to as RIB-A T and RIB-B T. Using a thermometer, the water temperature of the sampling sites was obtained. The pH of the sediments was measured with a pH electrode. Total nitrogen was quantified by the Nessler method [23] and total phosphorus was quantified by the Murphy-Riley method [24].

### 2.2. 16S rRNA and* amoA* Genes Libraries Construction

Environmental DNA was extracted from 0.5 g of lake sediment using the PowerSoil DNA isolation kit (MO BIO Laboratories, Inc.) according to the manufacturer's instructions. PCR primers 21f and 958r [25] were used to amplify the 16S rRNA gene using DNA from sediments samples as a template. For the* amoA* gene, the primers used were Arch amoAf and amoAr [26]. PCR assay conditions were the same as those originally described. The amplified DNA was visualized on agarose gels stained with ethidium bromide (10 mg/mL). The PCR-amplified DNA fragments were purified using the Wizard kit (SV Gel and PCR Clean-Up System, Promega) and cloned into the pGEM T easy (Promega) plasmid, according to the manufacturer's instructions. Recombinant plasmids were inserted into* Escherichia coli* calcium chloride-treated DH5*α* cells by heat shock treatment. The presence of the inserts was verified by selection of clones in LB agar plates supplemented with ampicillin (150 *μ*g/mL), IPTG (0.5 mM), and Xgal (0.00625%). Plasmid DNA was extracted with phenol-chloroform-isoamyl alcohol at 25 : 24 : 1 (vol/vol/vol). DNA was sequenced by the Sanger method at Macrogen Inc. (Korea). The sequences obtained were deposited in the GeneBank dataset under the accession numbers KF640285-KF640592 (16S rRNA gene) and KJ719076-KJ719244 (*amoA* gene).

### 2.3. Phylogenetic Analysis

The sequences from the 16S rRNA gene were used for comparative analysis with the Greengenes taxonomical database [27], using the Mothur software [28]. A threshold of 90% or higher identity with the database was used. Alignment of 16S rRNA and* amoA* gene sequences was performed with the ClustalX software [29].

Mothur was used to filter the gap columns generated by the alignment and was also used to generate the Ace, Chao, Shannon, and Simpson indexes. The 16S rRNA gene sequences were clustered with a sequence identity threshold of 97% for species, 95% for genus, 90% for class, and 80% for phylum [30]. Phylogenetic analysis of the* amoA* sequences was OTU based and the identity threshold considered was 90% [31]. Rarefaction curves were constructed with the Mothur software. The principal coordinate analysis (PCoA) was performed with the Unifrac program [32]. Sequences of the rRNA 16S gene from isolates of Euryarchaeota, Crenarchaeota, Thaumarchaeota, and Korarchaeota as well as sequences of uncultured* Archaea* found in the GenBank dataset were used to construct a phylogenetic tree with the MEGA software [33], using the maximum-likelihood method, a bootstrap value of 1,000 and the Tamura nucleotide substitution model.

## 3. Results and Discussion

All the sediments analyzed in the present study were 6 cm deep; therefore, they were part of the sediment layer most recently deposited [34]. All the samples presented slightly acidic pH values (Table 1), which may be due to hydrogen ion liberation in the medium caused by microbial metabolism and organic matter decomposition [10, 35]. Sediments obtained in the transition period (RIBT) had higher values of total nitrogen and phosphorus when compared to the samples obtained in the dry season (RIB D) (Table 1). As previously mentioned, environmental factors such as the occurrence of rain affect the nutrient content in lake sediments [11], which could explain the differences between the samples from each season. For the Cerrado soils it has already been shown that the changes of seasons affect the microbial community [36] and the present study suggests that these changes can also influence the microbial communities in freshwater lake sediments.

Four 16S rRNA gene clone libraries were constructed: two replicates for the dry season archaeal community (RIB-A D and RIB-B D) and two for the transition period between the dry and rainy seasons archaeal community (RIB-A T and RIB-B T). This resulted in a total of 308 sequences (75 sequences from RIB-A D, 70 sequences from RIB-B D, 75 sequences from RIB-A T, and 88 sequences from RIB-B T) with high Phred quality (>20) and size over 400 bp. Additionally, four archaeal* amoA* libraries were obtained from the same samples, resulting in 169 sequences with high Phred quality (>20) and size over 300 pb. Sample amoA-A D (RIB-A D) contains 43 sequences, amoA-B D (RIB-B D) 44 sequences, amoA-A T (RIB-A T) 42 sequences, and amoA-B T (RIB-B T) 40 sequences.

**Figure 1:**
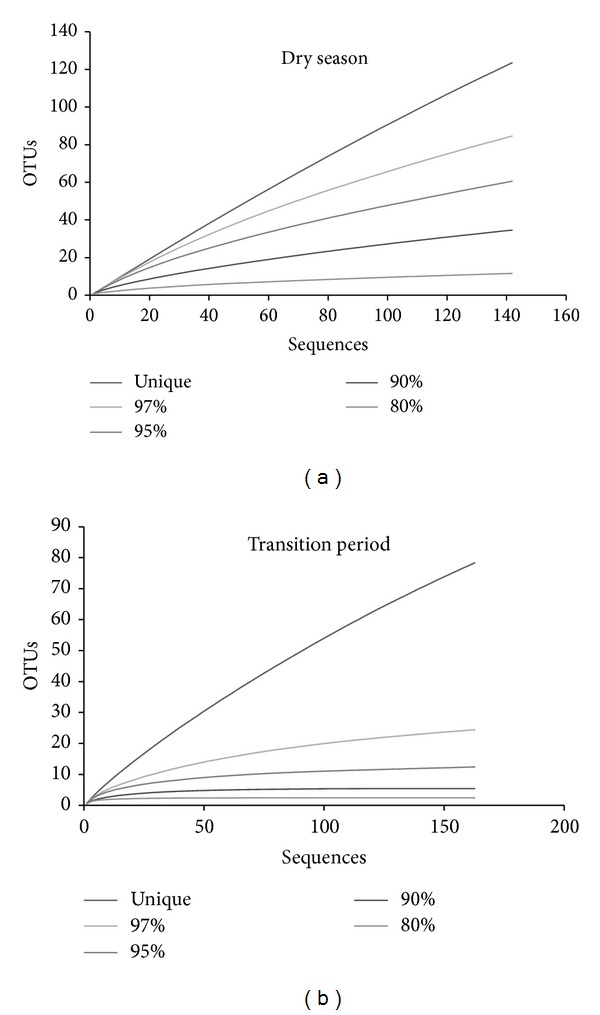
Rarefaction curves for archaeal 16S rRNA gene sequences from Cerrado freshwater lake sediments at different sequence identity of sequences obtained in the dry season (a) and in the transition period (b). Curves were obtained using the Mothur software.

Using sequence similarity of 97% or greater for the species level [30], our results revealed a large difference in the number of OTUs in samples from different seasons (Table 2). We were able to detect 85 OTUs in the dry season (RIB D) and 25 OTUs in the transition period between the dry and rainy seasons (RIB T), suggesting a higher richness in the dry season. This is further observed with the richness indexes, Ace and Chao, both of which estimated a higher number of predicted OTUs for the dry season. Both diversity indexes, Shannon and Simpson, also indicate a higher diversity for the sediment archaeal community found in the dry season. Similarly, the rarefaction curves (Figure 1) show a plateau for the transition period samples even with a 97% 16S rRNA gene sequence similarity level, while no plateau is reached for the sediment samples from the dry season, indicating higher richness and diversity in the dry season. Changes in microbial diversity are usually related to changes in nutrient availability caused by environmental factors, including the presence of nitrogen and phosphate fertilizers in soils and seasonal changes [37–44]. Based on the increase in total nitrogen detected in the transition period samples (Table 1), we can speculate that the reduced diversity observed in these samples was due to the higher nitrogen availability, which could have favored selected groups of organisms and was detrimental to others, as described in other studies [45].

**Figure 2:**
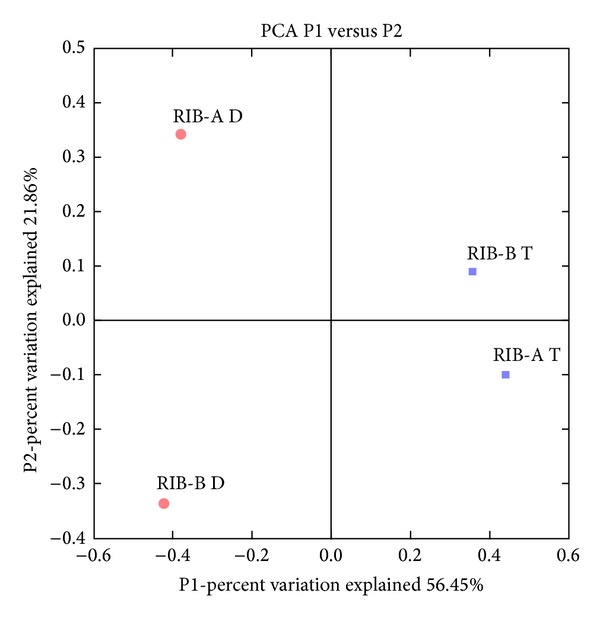
Unifrac principal coordinates analysis of Cerrado freshwater lake sediments archaeal 16S rRNA gene sequences obtained in the dry season (RIB-A D and RIB-B D) and transition between dry and rainy season (RIB-A T and RIB-B T).

The PCoA (Figure 2) was used to evaluate similarities between the communities of* Archaea* in the samples analyzed. This analysis shows that the replicates from the same season grouped closer, while samples retrieved in different seasons had significant differences, indicating a possible effect of the season change. Similar results were also obtained with the Libshuff test (data not shown). Most OTUs were shared between samples of the same season, while very few OTUs were shared between samples of different seasons (Table 3), further indicating two noticeably different community profiles. The overall decrease in diversity found in the transition period differs from other studies. A study from Cruz-Martínez et al. [43] indicated that rain had little impact on a California soil microbial community. However, there is a lack of microbial diversity studies on environments naturally characterized by dry and rainy periods and more studies are needed to fully understand the ecological dynamics in these kinds of environments.

**Figure 3:**
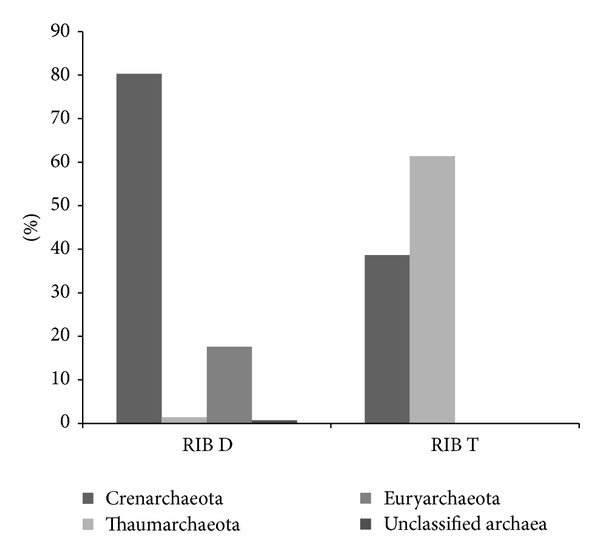
Histogram showing the taxonomic distribution of Cerrado fresh water lake sediment archaeal sequences obtained in the dry season (RIB D) and the transition between dry and rainy season (RIB T) among different phyla according to the Greengenes database.

**Figure 4:**
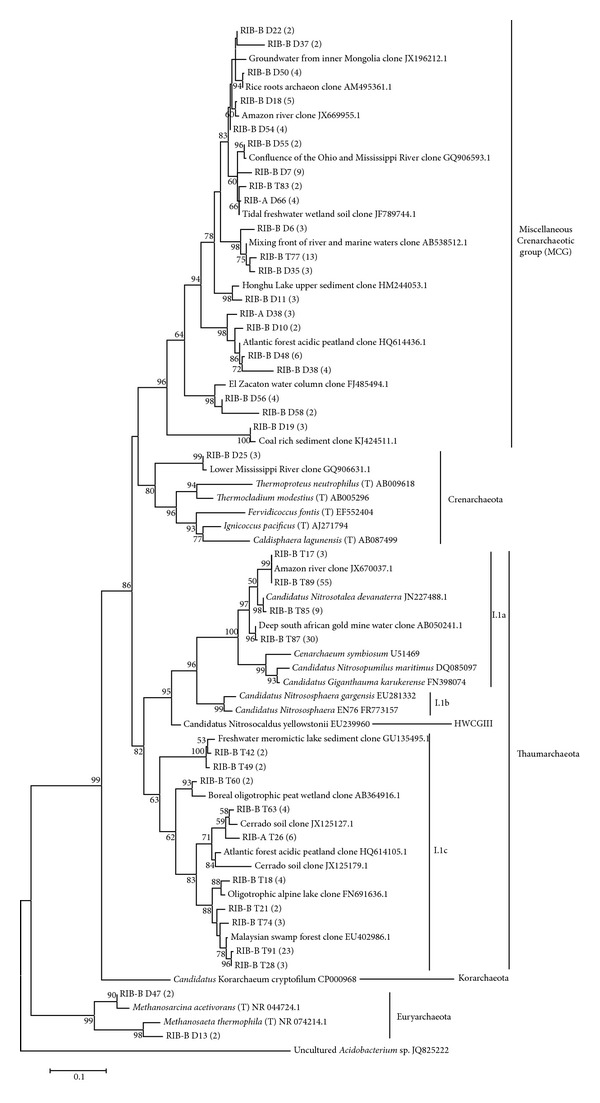
Phylogenetic tree of archaeal 16S rRNA gene OTUs (97%) obtained from Cerrado freshwater sediments in the dry season (RIB-A D and RIB-B D sequences) and in the dry to rainy season transition period (RIB-A T and RIB-B T sequences). The number of sequences that are represented by a specific node of the tree is indicated in parenthesis. The phylogenetic tree was inferred by the maximum-likelihood method using the MEGA software, bootstrap value of 1,000, and Tamura-Nei nucleotide substitution model. The percentage of bootstrap resamplings that supports each topological element is represented near the line. Reference sequences from different phyla were used for comparison and the bacterial sequence of the uncultured* Acidobacterium* sp. JQ825222 was used as an outgroup. Bootstrap values below 50 were not shown and singletons were not included.

According to the Greengenes taxonomical database, all 16S rRNA gene sequences were from the* Archaea* domain. One aspect worth mentioning is that, although this database considers Thaumarchaeota a class of the Crenarchaeota phylum, we classified Thaumarchaeota in the taxonomical level of phylum as suggested by Brochier-Armanet et al. [21]. The archaeal communities from the two seasons differed markedly in composition, even at phyla level (Figure 3). Out of 142 sequences, the dry season sample (RIB D) had predominantly crenarchaeotal sequences (80.28%). According to the database, 80.70% of the crenarchaeotal sequences were of the pGrfC26 order. This group is considered a part of the miscellaneous crenarchaeotic group (MCG) [46]. MCG* Archaea* can be found in diverse habitats, ranging from terrestrial to marine environments [47, 48]. However, this group is interestingly considered one of the most abundant groups in the sediment biosphere [49]. It is also important to note that the MCG group has no clear affiliation to any of the established archaeal phyla and has an unstable branching order in 16S rRNA trees [50]. There are currently no cultured isolates of this group and little is known about the roles of these organisms in biogeochemical cycles [46]. On the phylogenetic tree constructed with sequences representative of each OTU (97%) from both seasons (Figure 4), the MCG dry season sequences clustered in a group with only uncultured sequences retrieved from other studies, which was expected. It was also possible to detect in the dry season sequences classified as Euryarchaeota (17.60%) and 9 of them were affiliated with methanogenic groups. Methanogenic* Archaea* are commonly described in freshwater lake sediments [51–54], which have limited oxygen and high amounts of organic matter [55]. This can be also seen on the phylogenetic tree (Figure 4), where two dry season sequences (RIB-B D47 and RIB-B D13) aligned with methanogenic* Archaea*. The fact that the sediment layer analyzed was recently deposited may account for the low number of methanogenic* Archaea* found in our samples although primer bias cannot be ruled out [56, 57]. We were able to detect very few sequences classified as Thaumarchaeota (1.41%) or considered unclassified* Archaea* (0.71%) in the dry season samples (Figure 3).

Interestingly, among the samples of the transition period between the dry and rainy seasons (RIB T) we were able to detect predominantly sequences affiliated to the Thaumarchaeota phylum (61.35%) (Figure 3). All other sequences were affiliated to the Crenarchaeota phylum (38.65%). On the phylogenetic tree (Figure 4), the transition period sequences mostly clustered with the Thaumarchaeota phylum, the exception being sequences RIB-B T77 and RIB-B T83, which clustered with the MCG. The sequences RIB-BT 89 and RIB-BT87 represent a significantly higher number of sequences and were aligned within the I.1a cluster. This group is composed of organisms initially described in aquatic environments [58]. All other transition period sequences were part of the I.1c group, which contains organisms found in acidic soils [50]. It is known that Cerrado soils have acidic pH [2] and our group recently reported the presence of I.1c Thaumarchaota in these environments [8]. Given that only transition period sequences were found within this group and the fact that the sediments were retrieved near the margin, it is plausible that the rain caused leaching of the soils near the margin into the lake. We were not able to detect any members of the Euryarchaeota phylum in the transition period. It has been reported that ammonia oxidation is common among members of the Thaumarchaeota phylum [22, 59] and, in this sense, the detection of a great number of sequences affiliated to Thaumarchaeota might be correlated with the increase in total nitrogen in the transition period between the dry and rainy seasons samples (Table 1 and Figures 3 and 4). On the other hand, the absence of members of the Euryarchaeota phylum in sediments of the transition period could also be a consequence of methanogenesis inhibition mediated by nitrogen compounds [60, 61]. It is also worth mentioning that most uncultured sequences with high similarity to those found in the present study are from freshwater aquatic environments or soils with higher amounts of water (Figure 4).

Sequencing of* amoA* yielded 87 sequences from the dry season samples and 82 sequences from the transition period samples. *α*-Diversity analysis showed a higher number of* amoA* gene OTUs (Sobs) in the dry season (Table 4). This data is consistent with the higher diversity found for the 16S rRNA gene for the dry season. However, the 16S rRNA gene clone library failed to reveal this Thaumarchaeota diversity, since only 1.41% sequences from the dry season were considered of the Thaumarchaeota phylum. This result clearly highlights the importance of using more than one gene to describe specific microbial groups from natural environments [21]. Another factor that may account for the much reduced phylogenetic diversity of the* amoA* sequences is the fact that while our 16S rRNA gene analysis showed overall high diversity only four OTUs clustered in a confirmed ammonia-oxidizing group (I.1a) [21, 22] (Figure 4).

**Figure 5:**
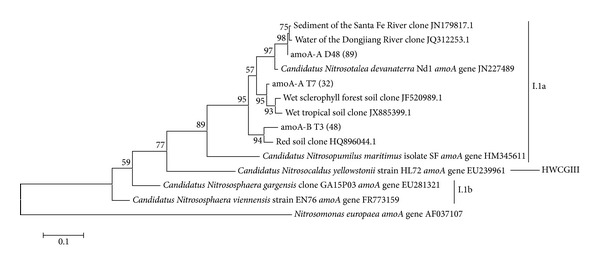
Phylogenetic tree of archaeal* amoA* OTUs (90%) obtained from Cerrado freshwater sediments in the dry season (amoA-A D and amoA-B D sequences) and in the dry to rainy season transition period (amoA-A T and amoA-B T sequences). The number of sequences that are represented by a specific node of the tree is indicated in parenthesis. The phylogenetic tree was inferred by the maximum-likelihood method using the MEGA software, bootstrap value of 1,000, and Tamura-Nei nucleotide substitution model. The percentage of bootstrap resamplings that supports each topological element is represented near the line. Reference sequences from different archaeal* amoA* genes were used for comparison and the bacterial sequence* Nitrosomonas europaea amoA* gene AF037107 was used as an outgroup. Bootstrap values below 50 were not shown and singletons were not included.

The* amoA* phylogenetic tree constructed (Figure 5) shows most of our sequences in the same cluster of* Nitrosotalea devanaterra*'s* amoA* gene. This is interesting, as the most abundant rRNA 16S gene sequences also clustered close to this organism (Figure 4).* N. devanaterra* was first described in acidic agricultural soils and was reported as a chemolithotrophic obligatory acidophilic soil ammonia oxidizer [62]. Similarly to our findings, a study on AOA diversity in high mountain lakes revealed sequences similar to* N. devanaterra* in aquatic environments [63] and suggested a phylogenetic conservation for the adaptive mechanisms to low pH environments [64]. However, the ecological role of* Archaea* in freshwater environments is still poorly understood [65–67]. As with the 16S rRNA phylogenetic tree, most* amoA* uncultured sequences with high similarity to ours were from environments with higher water content.

Although the number of samples analyzed imposes limitations, this study describes for the first time the diversity of* Archaea* in freshwater lake sediments of the Cerrado and provides evidence that rain can possibly affect archaeal diversity in these environments. This can be concluded because of the reduced diversity in samples from the transition period and markedly different archaeal community compositions between seasons. The events of resuspension and redeposition of the sediment surface that can be caused by rain alter nutritional composition (*i.e.,* nitrogen and phosphorus), which in turn can influence microbial communities. Future studies based on more sampling sites and use of quantitative methods will be performed to further investigate these events and better understand the ecological roles of* Archaea* in these environments.

## 4. Conclusions

We conclude that the occurrence of rain is likely a factor that influences the archaeal communities in freshwater lake sediments. We were able to observe higher richness and diversity of organisms in the dry season with a few sequences being from methanogenic members. The decrease of methanogenic* Archaea* in the transition period between the dry and rainy seasons could be due to an increase in nitrogen, which inhibits methanogenesis. The higher proportion of organisms from the Thaumarchaeota phylum could also be explained by this change in nutritional content. While in both seasons the presence of* amoA* genes was detected, a higher number of OTUs was observed in the dry season and in both cases these sequences were more similar to* Nitrosotalea devanaterra*'s* amoA* gene.

## Figures and Tables

**Table 1:** Comparison of physicochemical properties of freshwater lake sediments from replicates of the dry season (RIB D) and of the transition period between the dry and rainy seasons (RIB T).

	RIB D	RIB T
Sampling date	May, 2010	September, 2010
Water temperature (°C)	25.85 ± 0.35	21.60 ± 0.14
Depth (cm)	5.45 ± 0.07	4.70 ± 0.14
pH	5.57 ± 0.09	5.78 ± 0.20
P-total (*μ*g/L)	9.00 ± 1.41	18.50 ± 3.54
N-total (*μ*g/L)	11.00 ± 1.41	23.50 ± 2.12

**Table 2:** *α*-Diversity analysis for Cerrado freshwater lake sediment rRNA 16S gene sequences.

Season	Similarity (%)	Nseqs	Sobs	Chao	Ace	Shannon	Simpson	Coverage
RIB D∗	97	142	85	288.33	340.85	4.21	0.01	57.04
RIB T∗∗	97	163	25	31.00	34.36	2.28	0.17	94.47

*Dry season; ∗∗transition between dry and rainy seasons.

**Table 3:** Presence of the ten most abundant OTUs from each season among the lake sediments retrieved in the dry season (RIB-A D and RIB-B D) and the transition period (RIB-A T and RIB-B T).

	OTU Rep	RIB-A D	RIB-B D	RIB-A T	RIB-B T
Dry season	RIB-A D38	+	−	−	−
	RIB-B D11	−	+	−	−
	RIB-A D66	+	+	−	−
	RIB-B D50	+	+	−	−
	RIB-B D38	+	+	−	−
	RIB-B D54	+	+	−	−
	RIB-B D18	+	+	−	−
	RIB-B D56	+	+	−	−
	RIB-B D48	+	+	−	−
	RIB-B D7	+	+	−	+

Transition period	RIB-A T26	−	−	+	−
	RIB-B T18	−	−	+	+
	RIB-B T28	+	−	−	+
	RIB-B T63	−	−	−	+
	RIB-B T74	−	−	−	+
	RIB-B T77	+	+	−	+
	RIB-B T85	−	−	+	+
	RIB-B T89	−	−	+	+
	RIB-B T91	−	−	+	+
	RIB-B T87	−	−	+	+

**Table 4:** *α*-Diversity analysis for the *amoA* gene sequences.

Season	Similarity (%)	Nseqs	Sobs
amoA D∗	90	87	7
amoA T∗∗	90	82	3

*Dry season; ∗∗transition between dry and rainy seasons.
